# Protocol for a phase III pragmatic stepped wedge cluster randomised controlled trial comparing the effectiveness and cost-effectiveness of screening and guidelines with, versus without, implementation strategies for improving pain in adults with cancer attending outpatient oncology and palliative care services: the Stop Cancer PAIN trial

**DOI:** 10.1186/s12913-018-3318-0

**Published:** 2018-07-16

**Authors:** Tim Luckett, Jane Phillips, Meera Agar, Lawrence Lam, Patricia M. Davidson, Nicola McCaffrey, Frances Boyle, Tim Shaw, David C. Currow, Alison Read, Annmarie Hosie, Melanie Lovell

**Affiliations:** 1 Faculty of Health, IMPACCT (Improving Palliative, Aged and Chronic Care through Clinical Research and Translation Sydney), University of Technology Sydney (UTS), Level 7, 235 Jones St, Ultimo, (PO Box 123), Sydney, NSW 2007 Australia; 2 Tung Wah College, Mongkok, Kowloon, Hong Kong; 3 School of Nursing, Johns Hopkins University, Baltimore, MD USA; 4 Deakin Health Economics, Centre for Population Health Research, School of Health and Social Development, Deakin University, Geelong, VIC Australia; 5 The Mater Hospital, Cancer Care, Sydney, NSW Australia; 6 Faculty of Health Sciences, Charles Perkins Centre, The University of Sydney, Sydney, NSW Australia; 7 Department of Renal/Oncology, Gosford Hospital, Gosford, NSW Australia; 8 Department of Palliative Care, HammondCare, Greenwich Hospital, Sydney, NSW Australia

**Keywords:** Cancer, Pain, Guidelines, Implementation, Translation, Self-management, Patient education, Health professional education, Audit and feedback, Clinical change champions

## Abstract

**Background:**

Pain is a common and distressing symptom in people with cancer, but is under-recognised and under-treated. Australian guidelines for ‘Cancer Pain Management in Adults’ are available on the Cancer Council Australia Cancer Guideline Wiki. This study aims to evaluate the effectiveness and cost-effectiveness of a suite of guideline implementation strategies for improving pain outcomes in adults with cancer in oncology and palliative care outpatient settings.

**Methods:**

The study will use a stepped-wedge cluster randomised controlled design, with oncology and palliative care outpatient services as the clusters. Patients will be eligible if they are adults with cancer and pain presenting to participating services during the study period. During an initial control arm, services will routinely screen patients for average and worst pain over the past 24 h using a 0–10 numerical rating scale (NRS) and have unfettered access to online guidelines. During the intervention arm, staff at each service will be encouraged to use: 1) a patient education booklet and self-management resource; 2) an online spaced learning cancer pain education module for clinicians from different disciplines; and 3) audit and feedback of service performance on key indices of cancer pain screening, assessment and management. Service-based clinical change champions will lead implementation of these strategies.

The trial’s primary outcome will be the probability that patients initially screened as having moderate-severe (≥5/10 NRS) worst pain experience a clinically important improvement one week later, defined as  ≥ 30% reduction. Secondary outcomes will include patient empowerment and quality of life, carer experience, and cost-effectiveness. For the main analysis, linear mixed models will be used, accounting for clustering and the longitudinal design. Eighty-two patients per service at six services (*N* = 492) will provide > 90% power. A qualitative sub-study and analyses of structural and process factors will explore opportunities for further refinement and tailoring of the intervention.

**Discussion:**

This pragmatic trial will inform implementation of guidelines across a range of oncology and palliative care outpatient service contexts. If found effective, the implementation strategies will be made freely available on the Wiki alongside the guidelines.

**Trial registration:**

Registered 23/01/2015 on the Australian New Zealand Clinical Trials Registry (ACTRN12615000064505).

## Background

Despite of decades of research and practice improvement initiatives, 39–66% of people with cancer experience pain, and up to 40% of people with pain receive inadequate analgesia [1, 2]. Under-treated pain not only reduces patient quality of life (QOL) [3] but also increases health service use and costs [4]. Barriers to pain assessment and management occur at the levels of the patient (e.g. reluctance to report pain, misconceptions regarding opioids), clinician (e.g. lack of time and expertise), service (e.g. inadequate process for screening) and healthcare system (e.g. lack of coordination) [5–10]. Routine screening and implementation of evidence-based guidelines can improve quality of care and outcomes for cancer pain [11–13]. However, clinicians are unlikely to use symptom screening results or guidelines unless they are motivated and supported by focused strategies [14, 15].

The current authors have undertaken a program of work to develop and implement Australian guidelines for ‘Cancer Pain Management in Adults’, which are now available online via the Cancer Council Australia Cancer Guidelines Wiki [16, 17]. This program was structured in accordance with the UK Medical Research Council’s (MRC) Framework for complex interventions, defined as those with multiple interacting components that need tailoring to local healthcare settings and other contexts [18, 19]. The MRC recommends that complex intervention programs consist of four phases: development, feasibility and piloting, evaluation, and implementation. The *development phase* of our program involved a national survey of current practice [20–22], two systematic reviews [5, 23] and consideration of the wider literature by an Organising Committee within a theoretical framework called the Behaviour Change Wheel [24]. This process identified three strategies showing promise for improving care processes and outcomes for cancer pain: patient training, clinician education, and audit and feedback. These strategies are discussed in detail in the Methods section of the current paper. Our *piloting phase* tested the feasibility of screening for pain and auditing pain assessment and management in adults attending palliative care and oncology services [25]. The current paper focuses on the protocol for the *evaluation phase*, which aims to test the effectiveness and cost-effectiveness of all three guideline implementation strategies combined for improving pain outcomes in adults with cancer attending oncology and palliative care outpatient services.

## Methods/design

### Aims

#### Primary aim

The study’s primary aim is to evaluate capacity for a suite of three guideline implementation strategies to increase the probability that patients initially screened as having moderate-severe (≥5) worst pain on a 0–10 numerical rating scale (NRS) will experience a clinically important improvement of ≥30% one week later.

#### Secondary aims

Secondary aims are:

1. to evaluate capacity of the three guideline implementation strategies versus a control arm to:

i. reduce mean worst and average pain severity across all patients screened as having clinically relevant (≥2 NRS) or moderate-severe (≥5 NRS) worst pain from time of screening to one, two and four week later by half a standard deviation (0.5 SD);
ii. demonstrate a between-arm difference in patient empowerment in patients screened as having clinically relevant (≥2 NRS) worst pain, as measured by 0.5 SD difference on the Health Education Impact Questionnaire (heiQ) (41) at one, two and four weeks post-screening;
iii. demonstrate a between-arm difference in the experiences of unpaid carers of participating patients, as measured by a 0.5 SD difference on the Carer Experience Scale (CES) [26] at two and four weeks post-screening;
iv. demonstrate a between-arm difference on mean patient quality of life (QOL), as measured by a difference of 0.5 SD on the European Organisation for Research and Treatment of Cancer Palliative Care (EORTC QLQ C15-PAL) [27] at two and four weeks post-screening;

2. to evaluate the cost-effectiveness of the three guideline implementation strategies based on incremental cost per additional responder on the primary outcome;
3. to inform refinement and tailoring of the implementation strategies for different service settings.

### Design and setting

**Fig. 1:**
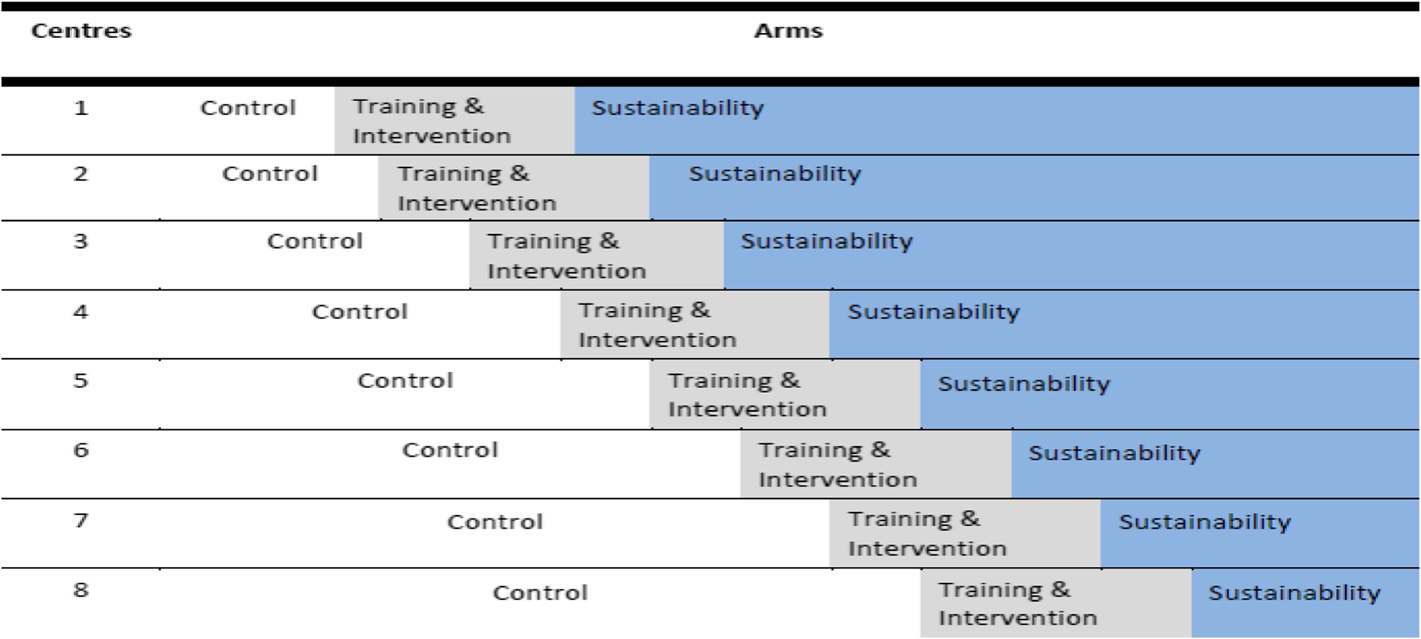
Stepped wedge design with staggered introduction of training/intervention in 8 services

The study will use a stepped-wedge cluster randomised controlled trial (RCT) design, with Australian oncology and palliative care services as the units of randomisation. In this design, randomisation concerns the sequence in which services transition between experimental conditions rather than the condition to which they are allocated (see Fig. 1). Each participating patient and carer will contribute data to only one arm, not both.

While needing a longer timeline than a parallel cluster design, a stepped-wedge design has the following advantages for translational research: 1) the design controls for between-service variation in baseline practice; 2) statistical power is boosted by the opportunity to assess intervention effects in a pre/post comparison across services; and 3) assessment of longitudinal effects (i.e. sustainability) can occur in services that transition earlier [28].

Ethics approval was granted by the South Western Sydney Local Health District Human Research Ethics Committee (HREC). Protocol modifications will be submitted to the HREC for approval, and modifications made to the trial registry information as needed.

This protocol complies with reporting requirements outlined in the cluster RCT extension of the Consolidated Standards of Reporting Trials (CONSORT) [29] and Standard Protocol Items: Recommendations for Interventional Trials (SPIRIT) [30].

### Participants

#### Services

Services will be selected to inform understanding of intervention effectiveness across services of differing geographic locations (e.g. state/territories; metropolitan, regional, rural) and configurations of oncology and palliative care. Study sites are included in the trial’s entry on the Australian New Zealand Clinical Trials Registry.

#### Patients

There will be two patient participant populations for this study. The first will contribute to primary outcome data, and the second to secondary outcomes. Because the study is funded by the National Breast Cancer Foundation (NBCF) of Australia, sampling will aim to recruit ≥60% patients with breast cancer. It is anticipated that breast cancer’s prevalence and this population’s proven willingness to participate in clinical trials [31] will make over-sampling unnecessary.

Participant eligibility criteria will follow recommendations from the Methods for Researching End of Life Care (MORECare) initiative [32] by targeting people most likely to benefit from the intervention. Inclusion criteria for patients to contribute to primary outcome will be: 1) attending a participating service as an outpatient during the study period; 2) having a diagnosis of advanced cancer of any type; 3) ability to self-complete a 0–10 NRS for severity of worst and average pain in English or one of five widely-spoken languages most commonly associated with poor English proficiency in Australia (Chinese, Italian, Greek, Vietnamese and Arabic) [33]; 4) choosing not to opt-out of being contacted one week later to complete the NRS and giving verbal informed consent to participate when contacted; and 5) a score of ≥5 on the NRS for worst pain. Inclusion criteria for patients contributing to secondary outcomes will be the same as criteria one, two, three and four above, as well as: 5) a score of ≥2 on the NRS for worst pain; and 6) spoken English proficiency sufficient to complete secondary outcome measures.

Patients will be excluded if they have already participated in the study either at another service taking part in the trial or at the same service when it was in the control arm, or are documented as having cognitive impairment that would preclude capacity to give informed consent.

Potentially eligible patients will be identified by the research team via a review of service pain screening data (see below), which will include information about their NRS score, whether they have opted out of being contacted, and their cancer status (completed by clinic staff). Patients will be telephoned at home, and the verbal informed consent procedure undertaken.

#### Carers

Unpaid carers will be eligible if they: 1) are identified by a patient who has given informed consent to participate in the study as providing them with substantial emotional and practical support in an unpaid capacity; 2) are willing to provide verbal informed consent to participate; and 3) have sufficient spoken English proficiency to complete a survey and/or interview.

Carers will be excluded if the patient for whom they provide care does not participate in any secondary outcome components.

#### Service staff

Service staff will be eligible to participate if they are: 1) employed on a permanent basis either full- or part-time at a participating service in a role that provides clinical care to patients with cancer pain or front desk administrative support; and 2) provide informed consent.

Service staff will be excluded if they are working at a participating service on a casual or agency basis.

### Intervention

Each participating service will first be included in the control arm and then transition to the intervention arm, defined as follows. Due to the pragmatic nature of the trial, no limit is placed on other interventions each service can undertake during the study period.

#### Control arm

We will compare the intervention with usual care plus the introduction of routine screening for pain where this is not already common practice. The rationale for including a screening system in the usual care arm is that this is already routine practice in some oncology and palliative care services across Australia.

Management and clinicians at participating services will be made aware of the Australian online guidelines for ‘Cancer Pain Management in Adults’ and the study’s aim to test implementation of these. Services that are already utilising an electronic or paper-based symptom screening system will be asked to incorporate the study’s NRS for worst and average pain over the past 24 h. Where no screening system is established, services will be equipped with a paper-based system for symptom screening and providing individual patient reports for treating clinicians to refer to during consultations. A paper-based rather than electronic screening system has been chosen because a pilot study conducted by the current authors found that implementing electronic screening can be resource-intensive and logistically challenging [25]. Acknowledging that migrants with limited proficiency in their host-country language may be at special risk of poor outcomes [34], screening measures will be made available in five widely-spoken languages most commonly associated with poor English proficiency in Australia (Chinese, Italian, Greek, Vietnamese and Arabic) [33].

While access to pain screening results and online guidelines will be unfettered, services will not be given strategies to encourage or support clinicians in using these.

The control arm at each service will continue as long as necessary to recruit the target sample size.

#### Intervention arm

At the beginning of a ***training phase***, two clinicians at each service will take on the role of ‘clinical change champions’ to lead local implementation of the intervention with support from the project team. Two are needed to cover any periods of absence. Selection of clinical change champions will be conducted in partnership with managers at each service. Champions can be of any discipline and role provided they meet established criteria [35].

Champions will attend training at their service aimed at tailoring the intervention to local needs and contexts. Champions will be involved in administering the audit tool and feeding back data to staff in order to identify strengths and weaknesses at their service against recommendations in the guidelines. They will also be supported to identify barriers and facilitators to cancer pain management and helped to develop solutions. Champions will be invited to attend monthly teleconferences with champions at other services to create a community of practice that will enable knowledge sharing, knowledge creation and identity building [36]. The degree to which champions take on all responsibilities or delegate these to other staff will be tailored to each service.

Also during the training phase, all clinical and reception-desk staff will be given an overview of the guidelines and implementation resources by the champions at their service with support from the project team.

During the training phase and for a period of three weeks afterwards, data will not contribute to outcome measurement to allow time for the intervention to become established as routine practice.

During the ***intervention phase***, the process for feeding back individual patient results from screening will be monitored, with medical teams receiving a summary report of screening data for each patient prior to consultation to inform management, and a copy filed in the medical record. The process by which this happens will vary across services according to local systems.

Three implementation strategies will be used to address commonly reported barriers and facilitators in the most parsimonious way. Findings from a systematic review suggest that coverage of barriers, rather than number of strategies, may be important in successfully implementing guidelines [37]. Therefore, one strategy was chosen to intervene at each of the levels of patient, clinician and health service, with strategies designed to address barriers across more than one level wherever possible. See Table 1 for mapping of the strategies against behaviour change ‘functions’ as identified by Michie et al.’s Behaviour Change Wheel [24].

**Table 1:** Behaviour change ‘functions’ (Michie et al., 2011 [24]) and related strategies employed to overcome barriers to cancer pain assessment and management in the Stop Cancer PAIN Trial (adapted from [68])

Behaviour change function	Strategies for overcoming barriers to cancer pain assessment and management		
	Patient level	Clinician level	Service/system levels
Education - Increasing knowledge or understanding	Information on types of pain, medications and side-effects (including low risk of opioid addiction) (OVERCOMING CANCER PAIN BOOKLET)	Information on opioid dosage, conversion and use in patients who are older and/or have renal failure (GUIDELINES, SPACED LEARNING)	Data on prevalence of cancer pain enabling comparison between services (AUDIT)
Persuasion - Using communication to induce positive or negative feelings or stimulate action	Goal setting, reflection on exacerbating/alleviating factors, management strategies, when/from whom to seek help aimed at reframing pain and promoting sense of control (SELF-MANAGEMENT RESOURCE)	Patient advocacy for person-centred care (SELF-MANAGEMENT RESOURCE) Local data on prevalence of cancer pain and performance on quality indicators (SCREEING, AUDIT)	Data on hospitalisations and other healthcare costs resulting from cancer pain (ECONOMIC EVALUATION)
Incentivisation - Creating expectation of reward/Coercion Creating expectation of punishment or cost	Goal setting, monitoring of pain (SELF-MANAGEMENT RESOURCE)	Quality improvement targets on pain assessment, management and outcomes (AUDIT)	
Training - Imparting skills	Skills development in rating pain severity and self-managing pain (SELF-MANAGEMENT RESOURCE)	Skill development in assessment, management and providing patient education (SPACED LEARNING, SELF-MANAGEMENT RESOURCE)	Development of service capacity to routinely screen for pain (SCREENING)
Environmental restructuring - Changing the physical or social context	Encouraging reporting of pain (SCREENING, SELF-MANAGEMENT RESOURCE)	Increased focus on cancer pain care (SCREENING, GUIDELINES, PATHWAY, AUDIT, SPACED LEARNING, SELF-MANAGEMENT RESOURCE)	
Modelling - Providing an example for people to aspire to or imitate	Personal stories of well managed pain (OVERCOMING CANCER PAIN DVD)	League table (SPACED LEARNING,) Modelling change (CLINICAL CHANGE CHAMPIONS)	Community of practice
Enablement - Increasing means/reducing barriers to increase capability or opportunity	Tools for promoting pain reporting, enhancing doctor/patient communication, and increasing priority/time accorded pain (SCREENING, SELF-MANAGEMENT RESOURCES)		Patient-held record supports information transfer and care coordination between providers (SELF-MANAGEMENT RESOURCES)

*The patient-level strategy* includes a written resource to help patients report and self-manage their pain, support coordination and communication between patients and clinicians, and advocate for evidence-based person-centred care [38]. This resource was designed for use in conjunction with an education booklet and DVD designed by members of the current team (ML, FB) called ‘Overcoming Cancer Pain’, published by Cancer Council Australia [39] and widely available in Australian oncology and palliative care services. The booklet includes information on pain, general advice on non-pharmacological and pharmacological management, and a pain diary template. The booklet and DVD were found to reduce pain versus usual care in a previous RCT [40]. The self-management resource includes: 1) a template for setting specific, measurable, achievable, relevant and time-bound (SMART) care goals, as well as identifying potential obstacles and ways to overcome these, and 2) an action plan detailing exacerbating and alleviating factors, current pharmacological and non-pharmacological management strategies, and contacts for support. Interviews with patients have provided preliminary support for the usefulness of the self-management resource [38, 41]. Patients and their physicians will set goals and complete the plan together to ensure shared understanding and decision-making. Patients will be encouraged to use the resource as a patient-held record that they can take to every healthcare appointment to support communication and ensure that care is coordinated and centred on their needs.

*The strategy selected for the clinician level* is a spaced learning training module for clinicians of different disciplines aimed at improving knowledge of guideline recommendations and applying this within the context of person-centred care. Spaced learning will be delivered online using the QStream Healthcare Solutions platform. The spaced learning format has been shown to significantly improve knowledge and retention of guideline content in RCTs [42]. This approach was chosen because it is a feasible form of education within busy clinical environments and is applicable to all members of multi-disciplinary teams. Questions testing knowledge of guideline recommendations are delivered to participants via email, which also provides immediate feedback about whether the questions have been answered correctly. Incorrect answers are resubmitted to participants at a later date and only retired when they have been answered correctly on two occasions. The use of clinical vignettes in each question ensures that learning is focused on the needs of an individual patient rather than only on biomedical aspects. Members of the current team (JP, TS) have developed and piloted a spaced learning module for cancer pain assessment in the palliative care setting, where it was associated with increased levels of nurse knowledge and documentation of pain, as well as reduced pain severity for patients [43, 44]. Further modules are being developed by the team to test and improve clinician understanding of cancer pain assessment and management.

At the service level, an audit and feedback mechanism has been chosen as the best strategy following evidence from a Cochrane systematic review that this strategy can lead to improvement in provider compliance with desired practice [45], including implementation of cancer pain guidelines [46]. The team adapted an audit tool developed by Victoria Health, which collects data on key indices of cancer pain care, including screening, assessment, regular and breakthrough analgesia, and management of adverse effects [47]. During the pilot phase of this program, the audit tool was found feasible to implement, and changes were made to improve inter-rater reliability and relevance to the current intervention [25]. In accordance with best-practice, feedback will be provided in written (e.g. newsletter) as well as verbal formats and accompanied by discussion aimed at setting and meeting SMART targets via an agreed practice improvement plan [48]. Discussions will be framed within a Plan-Do-Study-Act (PDSA) model for quality improvement, with which Australian clinicians are familiar [49].

During a ***sustainability phase***, service clinicians who are trained in the use of the audit tool will provide the research team with de-identified audit data on pain assessment and management for patients screened as having a severe (≥7 NRS) pain score to assess whether adherence to guideline recommendations has continued.

### Outcomes

The study period for each patient will be four weeks or until death, whichever is the shorter time. See Table 2 for the enrolment and assessment schedule.

**Table 2:** Enrolment and assessment schedule for patient participants in the Stop Cancer PAIN Trial

	STUDY PERIOD				
	Allocation	*Post-allocation*			*Close-out*
TIMEPOINT	0	*Week 1*	*Week 2*	*Week 4*	*t*_*x*_
ENROLMENT:					
Eligibility screen	X				
Informed consent	Opt-out	X			
Allocation	X				
ASSESSMENTS:					
Patient worst/average pain severity	X	X	X	X	
Patient quality of life		X	X	X	
Patient empowerment		X	X	X	
Carer experience			X	X	
Patient and carer interviews				X	
Patient MBS/PBS data					X

*MBS* Medicare Benefits Schedule, *PBS* Pharmaceutical Benefits Scheme

#### Primary outcome

The primary outcome will relate to both individual patient and cluster levels and concern the probability that patients at each service initially screened as having moderate-severe (≥5 NRS) worst pain will experience a clinically important improvement one week later, defined as a ≥ 30% reduction in their original rating.

One week following positive screen is considered sufficient time to allow for comprehensive assessment and any new treatment regimens to become established. Pain over the past 24 h was chosen to balance the need to avoid recall bias with sampling a representative time window. The NRS is the optimal brief measure of pain severity based on compliance rates, responsiveness, ease of use and applicability [50]. The NRS is also the response option used by most widely used and validated multi-dimensional pain scales (e.g. Brief Pain Inventory [51]). A rating of 5 on a 0–10 NRS has become established as the threshold for moderate cancer pain [52]. Patients with pain of this magnitude or higher will form the focus of the primary endpoint because of the added urgency of reducing moderate-severe pain versus mild.

A 30% reduction in pain was selected as the minimal clinically important difference (MCID) based on recommendations for pain trials by the US Food and Drug Administration, which takes into account evidence that the number of NRS points needed for a MCID varies according to the baseline score [53].

To minimise selection bias, the study will use an opt-out procedure for patients’ permission to obtain their contact details and telephone them one week later to measure the primary outcome. Upon contact, patients will be invited to give verbal informed consent to provide primary outcome data, and will remain at liberty to refuse at this time.

**Fig. 2:**
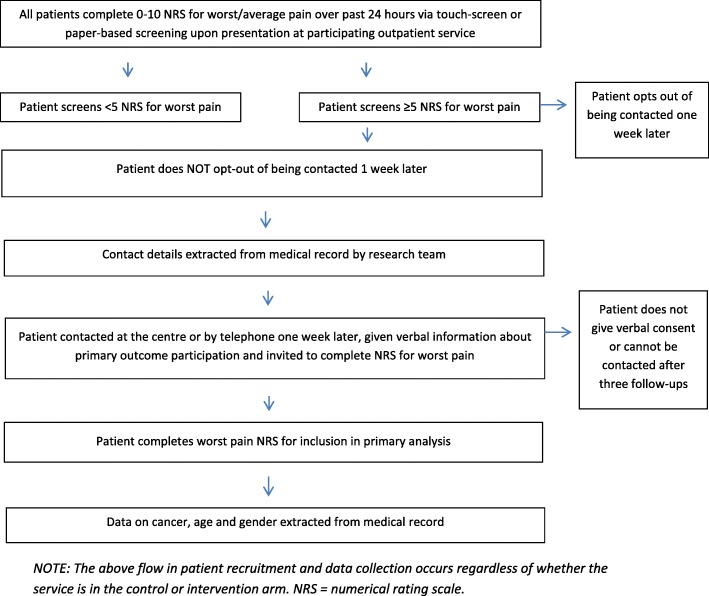
Study diagram of patient recruitment and data collection for primary endpoint of worst pain over past 24 h at one week after screening with worst pain of ≥5 on a 0–10 numerical rating scale (NRS)

For a flow diagram of recruitment of patients to the primary outcome component of this study, see Fig. 2.

#### Secondary outcomes

Secondary outcomes will assume a MCID to be 0.5 standard deviation (SD), using an established rule of thumb for patient reported outcomes [54].

##### Patient pain

Secondary outcomes for pain will be at the cluster level and relate to the potential for the intervention versus control arm to result in: 1) mean reduction for worst pain severity in patients with clinically significant (≥2 NRS) and moderate-severe (≥5 NRS) worst pain from screening to one, two and four weeks later; and 2) mean reduction for worst and average pain across all screenings, to give a population measure of effect.

A waiver on patient consent has been authorised by the HREC to include de-identified pain screening data from all patients attending services during the control and intervention arms to avoid selection bias. However, informed consent will be required for participation in all other secondary outcomes, detailed as follows. The HREC has approved a verbal (rather than written) consent procedure for all outcomes except health service utilisation, for which written informed consent will be required using a standardised form developed by Medicare.

##### Patient quality of life

Patient QOL will be measured at weeks one, two and 4 using the EORTC QLQ C15-PAL [27], a short-form of the EORTC QLQ-C30 [55], which is among the most widely used cancer-specific QOL measures. The QLQ C15-PAL includes 15 items to reduce burden and focus on issues most relevant to people with advanced cancer. It provides scores for global QOL, physical functioning, psychological functioning, and symptoms that include fatigue, nausea and vomiting, breathlessness, insomnia, appetite loss and constipation. Importantly for this study, it also provides a score for pain interference and uses a recall period of the past week; both features will complement the two pain NRS, which focus on pain severity over the past 24 h. In this study, five additional items from the QLQ-C30 will be added to the C15-PAL at every administration to enable cost-utility evaluation using the QLQ-Utility measure [56]. These items assess physical functioning, role functioning, social functioning (two items) and diarrhea.

A MCID of 0.5 SD difference between control and intervention arms will provide a convenient single estimate that approximates to scale-specific MCIDs developed for the QLQ C15-PAL [57].

##### Patient empowerment

The Health Education Impact Questionnaire (heiQ) will be administered at weeks one, two and 4 to evaluate patient empowerment. A version of the heiQ developed in Australia specifically for cancer will be used. The study includes scales measuring health service navigation, constructive attitudes and approaches and skill and technique acquisition [58]. As well as offering an index of self-reported self-management efficacy, it is hypothesised from our systematic review that patient empowerment may have a direct impact on pain experience itself [23].

##### Carer experience

The Carer Experience Scale (CES) will be administered at weeks two and 4 to evaluate the intervention effect on unpaid carers in terms of their relationship with patients, support received and ability to carry on with their own lives [26].

##### Structural and process measures

The implementation strategies evaluated by this study represent a complex intervention involving multiple interacting components [18]. As such, measures of infrastructure and processes for care are needed to assess fidelity of implementation, clarify causal mechanisms and identify influential contextual factors to inform further refinement and tailoring to different settings.

Service-level process measures are: screening rates, clinician training (via spaced learning and other initiatives), adherence to the guidelines, and documentation quality.

Staff-level process measures are: the amount of time spent in training and on cancer pain screening, assessment and management by clinical staff and front desk administrative staff. Staff will be invited to complete an anonymous online survey that asks them their role at the service and three separate questions on how much time they spent on cancer pain screening, assessment and management during their last complete work day.

Patient-level process measures are: number of consultations, multi-disciplinary input, primary care support, and indices of care recommended by the guidelines. Items of particular interest will be instances of screening, assessment, patient education, regular analgesia, breakthrough analgesia and management of side-effects (preventative and treatment). The audit tool (administered by trained personnel to standardise between arms and services) will assess participants’ clinical records retrospectively. Because it is often not documented in medical records, patients’ receipt of pain education will be measured by asking the patient at each time-point they contribute to secondary data collection.

##### Descriptive/control variables

Patient variables will include demographics, details of cancer diagnoses, performance status (Australia - modified Karnofsky Performance Status [AKPS] [59] at palliative care services and the Eastern Oncology Cooperative Group Performance Status [ECOG] [60] at oncology services) and comorbidities that may cause pain or influence its management (e.g. renal impairment). Carer variables will include demographics and relationship to the patient. Staff variables will be limited to role.

#### Qualitative sub-study

A sample of patients and carers (*n* = 20 respectively) who participate in secondary outcome measurement will be invited to take part in interviews 4 weeks after initial screening. Interviews will use a focused question route and ask about informants’ experiences of pain assessment and management, self-management education, and perceived person-centredness and coordination of care.

On completion of the intervention arm at each service, clinicians and front desk administrative staff will be invited to participate in interviews or focus groups to explore perceived barriers and facilitators to the intervention.

#### Data management

A designated staff member at each site will enter baseline screening data into an Excel spreadsheet specifically developed for this study. Participants will be identified by a study number only, and data will be stored in a locked filing cabinet or on password-protected computers at the coordinating site. The investigators and sponsor will have access to the final dataset. Because the intervention arm is aimed at encouraging clinicians to provide evidence-based care rather than use a novel intervention, no data monitoring committee is required. There are no plans for independent trial audit. In the event that unexpected adverse events are associated with the conduct of the trial, incident reports will be completed by the project team and updates provided to the HREC. Participant information sheets will provide contact details for complaints. Trial results will be communicated to the research and clinical communities via journal publication and conference presentation, to which the investigators and (where appropriate) representatives from participating services will contribute as authors.

### Sample size

To assess the appropriate study sample size and compute study power, we used a computer simulation allowing for 20% dropout by both services and patients. Drawing on data from 1612 consecutive patients [61], outcome data were generated to mimic pain scores we expect to see in the presenting population. We generated data from a beta distribution (scaled 0 to 1), rescaled to lie between 0 and 10, and then rounded to integer values. We then discarded data points < 2 to represent the sample of interest. We allowed service-specific means to vary between 0.05 and 0.15 to generate an appropriate intra-class correlation. We used a standard MCID of 0.5 effect size [62]. To more closely represent reality, we allowed for variation in the responses of each patient to intervention. Once a hypothetical study population had been generated, we ran a linear mixed model that included an intervention effect as well as an intervention by time effect, as well as a random service effect. We repeated this whole process several hundred times and then estimated power by computing the proportion of times that the null hypothesis would be rejected. We did this both for the main intervention effect, as well as the intervention by time effect. Two-sided hypothesis tests with type I errors of 0.05 were used. Assuming 82 patients per service at six services (*N* = 492), our study will have > 90% power to detect the main effect. The study will oversample by two services (i.e. a total of 8 services) and 18 patients per service (i.e. *n* = 100 at each service) to allow for drop-out.

### Randomisation

Randomisation will be conducted at a central office by a statistician (LL) not involved in the operation of the study. A random sequence will be generated using a computerised randomisation algorithm. Allocation will be based on clusters rather than individuals, and randomisation will be concerned with the time at which each service transitions from the control arm to training and intervention. Randomisation will occur prior to data being collected at each service. Due to the nature of the intervention, concealment of allocation is not feasible. Stratification will be unnecessary because each service will serve as its own control. Patients will be automatically allocated to control or intervention arms according to the current arm of their treatment service upon their first presentation.

Randomisation will be conducted by the trial statistician (LL). Services will be enrolled by members of the project team prior to randomisation after a contract is signed by service managers. No concealment will be feasible for managers and clinicians due to the nature of the trial design and intervention. Because randomisation is concerned with the time at which each service will commence the intervention, individual patients and caregivers will be enrolled after randomisation but will be unaware of service allocation at the time of their enrolment.

### Blinding

The nature of the intervention in this study renders blinding of clinicians impractical, and previous research suggests that uptake of guidelines in the control arm will be unlikely in the absence of targeted strategies of the kind to be introduced in the intervention arm [14]. Information for patients will provide only general information about the aims of the study (i.e. that it will compare different approaches to cancer pain management) rather than specifics of the design and intervention. Previous experience of cluster RCTs by the current team suggests that attempts to blind research assistants collecting data will be impractical. Instead, attention will be paid to research assistant training and standardisation of data collection as ways to limit the potential for bias. Personnel conducting analyses will be blinded to service allocation.

### Statistical methods

For the main analysis, linear mixed models [63] will be used to model the outcomes of interest, while accounting for the clustering and longitudinal design. We have conducted computer simulations to confirm that this approach will work well for pain NRS scores measured on an eleven point scale. The linear mixed modeling framework is very flexible. For example, it will allow for testing whether treatment effects diminish over time. It will also allow the incorporation of additional covariates of interest, for instance patient age, gender, ethnicity and factors related to disease; and inclusion of covariates that reflect characteristics of the study services. Statistical analysis will be performed using appropriate software capable of handling data from this study design. Analyses will be repeated for all patients and patients newly referred during the intervention arm to control for the possibility that outcomes are influenced by care received prior to the intervention being implemented. For secondary patient outcomes, analyses will be repeated for patients with clinically relevant (≥2 NRS) and moderate-severe (≥5 NRS) worst and average pain.

### Economic analysis

An economic evaluation will estimate the incremental costs and consequences of the intervention versus control arm. The primary outcome for the cost-effectiveness analysis will be the incremental cost per additional responder, with response defined as a clinically important improvement of 30% on a 0–10 NRS one week post-screening for those with moderate-severe (≥5 NRS) worst pain). The incremental cost per additional quality adjusted life years (QALY) will be the secondary outcome. Utility scores will be derived from the EORTC QLQ-C15-PAL and 5 additional items from the QLQ-C30 (mapped from QLQ-Utility [56]) and index values for carers from the CES. Survey responses will be linked to health service utilisation data accessed through the Centre for Health Record Linkage (CHeReL) and from Commonwealth datasets (Medicare Benefits Scheme (MBS); Pharmaceutical Benefits Scheme [PBS]) and hospital records. Variables will include: Australian Refined Diagnosis Related Groups (ARDRG) code; number of hospitalisations; length of hospital stays; number of emergency department, outpatient, psychology/psychiatry and general practitioner (GP) visits; health insurance status; medication usage. Costs of the intervention will relate to the screening systems implemented at each service, materials for training, and staff time spent on attending training and screening, assessing and managing cancer pain. As economic data may be skewed, confidence intervals will be estimated with bootstrap methods [64]. Sensitivity analysis will examine the effect of assumptions and determine which cost components drive the results.

### Qualitative analysis

Interview and focus group data will be transcribed verbatim and imported into NVivo 11 software for analysis. Analysis will use an integrative qualitative method designed specifically for informing health service interventions [65]. This method uses both deductive and inductive approaches to ensure themes build on previous research whilst also remaining open to new insights from participants’ experiences. To reduce risk of bias and enrich interpretation, analysis will be conducted by two independent researchers who will then meet to agree themes. As well as coding concepts (concept codes), researchers will also code relationships between concept codes (relationship codes), participants’ positive or negative appraisals (participant perspective codes) and relevant variables relating to participant characteristics (participant characteristic codes) and service (service codes). Preliminary themes will be subject to review by the larger research team before finalizing the code structure.

The Promoting Action on Research Implementation in Health Services (PARiHS) framework will be applied to analyses of interview/focus group data and the structural and process measures outlined above to consider the influences of evidence, context and facilitation on the success of the intervention [66].

## Discussion

The protocol for the Stop Cancer PAIN Trial has been designed to balance the need for rigorous evaluation with the flexibility required by complex interventions [67]. The proposed intervention is intended to take a coordinated approach to overcoming barriers to guideline implementation at system, provider and patient levels in order to ensure that care for cancer pain meets a best-practice standard across each service but can also be tailored to the needs of individual patients and families.

One of the advantages of a stepped wedge design over parallel cluster RCTs is that a diversity of services can be included without compromising comparability because each cluster serves as its own control. Inclusion of heterogeneous services in the proposed study should enable generalisability of results to a large proportion of Australian oncology and palliative care services. Our qualitative sub-study and analyses of structural and process factors will provide useful insights into the requisite conditions for the intervention to succeed and modifications needed for different service models.

The study’s methods are limited by a lack of blinding, which is necessitated by the nature of the design, intervention and outcomes. Standardised procedures for eligibility screening are designed to reduce the risk of selection bias that might arise from clinician and researcher knowledge of allocation. Logistic barriers will mean that sustainability assessment beyond the intervention arm period will be limited to audit data at the service level, rather than outcome measurement at the patient level. Sustainability of effects on patient and carer outcomes, therefore, will at best be inferred rather than directly observed. There is also a risk that practical issues relating to service availability and recruitment may make the randomisation schedule difficult to adhere to with regard to the order in which each cluster commences the control arm and transitions to the intervention [68]. The main purpose of the randomisation is to ensure that not all services commence at the same time and so limit any confounding that might occur from changes at the national level (e.g. policy) that coincide with transition from control to intervention arms. The precise order in which services commence and transition may be of lesser concern in terms of likely bias.

If implementation strategies are found to be cost-effective, they will be made available free-of-charge alongside the Australian guidelines for 'Cancer Pain Management in Adults' on the Cancer Council Australia Cancer Guidelines Wiki [16] to facilitate nationwide translation. In keeping with the continuous cycle recommended for complex interventions by the MRC, the Wiki platform will enable ongoing development and incorporation of changing evidence over time.

## Other information

### Registration

The trial was prospectively registered on the Australian New Zealand Clinical Trials Registry on 23rd January 2015 with the trial identification ACTRN12615000064505; World Health Organisation unique trial number U1111–1164-4649. The primary sponsor is The University of Sydney. TL is the contact for public and scientific queries: Tim Luckett; T + 61 2 9514 4861; E tim.luckett@uts.edu.au

### Protocol version number and date

I001/V 5, 5th April 2017.

### Roles and responsibilities

Decisions for the conduct of the trial are made by the Stop Cancer PAIN Trial Executive Committee (ML, TL, MA, JP). The trial is coordinated from IMPACCT (Improving Palliative, Aged and Chronic Care through Clinical Research and Translation) from the University of Technology Sydney. An Advisory Group meets twice yearly to identify synergies with other initiatives across Australia.

## Abbreviations

AKPS: Australia - modified Karnofsky Performance Status
ARDRG: Australian Refined Diagnosis Related Groups
CES: Carer Experience Scale
CHeReL: Centre for Health Record Linkage
CONSORT: Consolidated Standards of Reporting Trials and
ECOG: Eastern Oncology Cooperative Group Performance Status
EORTC QLQ C15-PAL: European Organisation for Research and Treatment of Cancer Palliative Care
heiQ: Health Education Impact Questionnaire
HREC: Human Research Ethics Committee
MBS: Medicare Benefits Scheme
MCID: Minimal clinically important difference
MORECare: Methods for Researching End of Life Care
MRC: (UK) Medical Research Council
NBCF: (Australian) National Breast Cancer Foundation
NRS: Numerical rating scale
PAIN: Pharmacological management, anti-cancer therapy, interventional therapy for non-responsive severe pain, non-pharmacological management
PARiHS: Promoting Action on Research Implementation in Health Services
PBS: Pharmaceutical Benefits Scheme
PDSA: Plan-Do-Study-Act
QALY: Quality adjusted life years
QOL: Quality of life
RCT: Randomised controlled trial
SD: Standard deviation
SMART: Specific, measurable, achievable, relevant and time-bound
SPIRIT: Standard Protocol Items: Recommendations for Interventional Trials
